# Identification of α-Glucosidase Inhibitors from *Scutellaria edelbergii*: ESI-LC-MS and Computational Approach

**DOI:** 10.3390/molecules27041322

**Published:** 2022-02-16

**Authors:** Muddaser Shah, Hazir Rahman, Ajmal Khan, Shabana Bibi, Obaid Ullah, Saeed Ullah, Najeeb Ur Rehman, Waheed Murad, Ahmed Al-Harrasi

**Affiliations:** 1 Department of Botany, Abdul Wali Khan University Mardan, Mardan 23200, Pakistan; muddasershah@awkum.edu.pk; 2 Natural and Medical Sciences Research Center, University of Nizwa, P.O. Box 33, Birkat Al Mauz, Nizwa 616, Oman; ajmalkhan@unizwa.edu.om (A.K.); obaidullah@unizwa.edu.om (O.U.); saeedullah@iccs.edu (S.U.); 3 Department of Microbiology, Abdul Wali Khan University Mardan, Mardan 23200, Pakistan; hazirrahman@awkum.edu.pk; 4 Yunnan Herbal Laboratory, College of Ecology and Environmental Sciences, Yunnan University, Kunming 650091, China; shabana_bibi@ynu.edu.cn; 5 International Joint Research Center for Sustainable Utilization of Cordyceps Bio Resources in China and Southeast Asia, Yunnan University, Kunming 650091, China; 6 Department of Chemistry, University of Malakand, Chakdara 18800, Pakistan; 7 H.E.J. Research Institute of Chemistry, International Center for Chemical and Biological Science, University of Karachi, Karachi 75270, Pakistan

**Keywords:** *S. edelbergii* Rech. f, in-vitro diabetic, ESI-LC-MS, computational analysis, intermolecular interactions, α-glucosidase

## Abstract

The recent study investigated the in vitro anti-diabetic impact of the crude extract (MeOH) and subfractions ethyl acetate (EtOAc); chloroform; n-butanol; n-hexane; and aqueous fraction of *S. edelbergii* and processed the active EtOAc fraction for the identification of chemical constituents for the first time via ESI-LC-MS analysis through positive ionization mode (PIM) and negative ionization mode (NIM); the identified compounds were further validated through computational analysis via standard approaches. The crude extract and subfractions presented appreciable activity against the α-glucosidase inhibitory assay. However, the EtOAc fraction with IC_50_ = 0.14 ± 0.06 µg/mL revealed the maximum potential among the fractions used, followed by the MeOH and n-hexane extract with IC_50_ = 1.47 ± 0.14 and 2.18 ± 0.30 µg/mL, respectively. Moreover, the acarbose showed an IC_50_ = 377.26 ± 1.20 µg/ mL whereas the least inhibition was observed for the chloroform fraction, with an IC_50_ = 23.97 ± 0.14 µg/mL. Due to the significance of the EtOAc fraction, when profiled for its chemical constituents, it presented 16 compounds among which the flavonoid class was dominant, and offered eight compounds, of which six were identified in NIM, and two compounds in PIM. Moreover, five terpenoids were identified—three and two in NIM and PIM, respectively—as well as two alkaloids, both of which were detected in PIM. The EtOAc fraction also contained one phenol that was noticed in PIM. The detected flavonoids, terpenoids, alkaloids, and phenols are well-known for their diverse biomedical applications. The potent EtOAc fraction was submitted to computational analysis for further validation of α-glucosidase significance to profile the responsible compounds. The pharmacokinetic estimations and protein-ligand molecular docking results with the support of molecular dynamic simulation trajectories at 100 ns suggested that two bioactive compounds—dihydrocatalpol and leucosceptoside A—from the EtOAc fraction presented excellent drug-like properties and stable conformations; hence, these bioactive compounds could be potential inhibitors of alpha-glucosidase enzyme based on intermolecular interactions with significant residues, docking score, and binding free energy estimation. The stated findings reflect that *S. edelbergii* is a rich source of bioactive compounds offering potential cures for diabetes mellitus; in particular, dihydrocatalpol and leucosceptoside A could be excellent therapeutic options for the progress of novel drugs to overcome diabetes mellitus.

## 1. Introduction

The genus *Scutellaria* L. (Lamiaceae) includes over 350 species and is extensively part of the conventional traditions for ailment purposes and is distributed mostly over mountain trails in the moderate hot and humid regions of America, Europe, and East Asia [1]. In Pakistan, the species of *Scutellaria* are found in the northern areas of Swat and Chitral. The genus *Scutellaria* has diverse biomedical applications due to its bioactive constituents, the screening of which commenced in 1889, and scutellarin (a flavone) from *Scutellaria altissima* was isolated for the first time in 1910 by Goldschmiedt and Lerner [2].

Around 295 chemical constituents in around 35 species of the genus *Scutellaria* have been isolated to date and have a significant role in the production of novel drugs. Dominant among them are flavonoids, terpenoids, alkaloids, steroids, and glycoside [1]. Due to its abundance of flavonoids, including baicalin, baicalein, and wogonin, and terpenoids comprising jodrellin A, jodrellin B, scutalbin A, and scutecyprol B, *Scutellaria* shows substantial value as a source of remedies such as antimicrobial, anti-inflammatory, and analgesic agents for various recent health complications [3]. Some species were also reported as prospective sources of agents that resist microbes, scavenge free radicals, inhibit inflammation, and relieve pain [3], and have been reported locally as well for their anti-diabetic significance [4].

Diabetes mellitus (DM) is a challenging disease related to insulin secretion, which is responsible for the balance of glucose in the blood and mainly regulated by flavonoids and terpenoids with the addition of alkaloids and steroids, which normalize active size responsible for insulin production and help cope with DM [5]. One of the most severe consequences of DM is hyperglycemia, which leads to long-term health complications, including cardiovascular diseases, metastatic cancer retinopathy, and nephropathy [1]. The prevalence rate varies over the globe; in Pakistan, the prevalence rate of diabetes type II (T2DM) is about 11.77 %, reflecting a high disease burden and increasing prevalence rate [2]. According to global estimates, it is predicted that some factors such as population growth, aging, urbanization, and lifestyle will lead to an increase in the global prevalence of DM, to 54% by the year 2030 [3]. α-glucosidase has become a valid target for drug design and problems related to hyperglycemia. Therefore, α-glucosidase inhibitors (AGIs) have displayed noteworthy consideration for clinical use in the treatment of hyperglycemia and, as well, use as antiviral agents [4]. Overcoming hyperglycemia by lowering the digestion and absorbance of carbohydrates is a key therapeutic strategy insight into the significant function of α-glucosidase. Hence, it is required to introduce new AGIs with relatively few side effects compared to the available clinical AGIs such as acarbose, voglibose, and miglitol [5,6].

As mentioned, some species of *Scutellaria* are used by local practitioners for blood cleansing and the treatment of inflammation, pain, and DM [3]. Thus, this study aimed to screen their antidiabetic potential, profile the active extracts via liquid chromatography-mass spectrometry to identify promising compounds for the treatment of various health complications, validate scientifically their local antidiabetic potential through in vitro antidiabetic assays, submit the most effective fractions to computational analysis for further validation of promising compounds, and, through isolation of the constituents considered useful for the treatment of the above-mentioned disorders, provide isolation experts and pharmacists with bioactive compounds for antidiabetic therapy.

## 2. Result and Discussion

The prepotency of medicinal plants is ratified through their bioactive constituents, which directly or indirectly depend upon edaphic and climatic characteristics, the quality and availability of water, and numerous other factors. In this ongoing study, *S. edelbergii* in crude extract and subfractions was screened for its in vitro antidiabetic capacity, and the most active fraction was submitted for qualitative assessment of its phytoconstituents through ESI-LC-MS analysis to highlight the active compounds, which were further subjected to computational screening to authenticate their anti-diabetic significance for scientific validation and contribute to the literature on *S. edelbergii* and its constituents as therapeutic agents. The selected genus had been reported earlier in the literature as a rich source of flavonoids, terpenoids, alkaloids, glycosides, and tannins [2]. The flavonoids, terpenoids, alkaloids, steroids, tannins, and phenols are well-known for their diverse biomedical applications [6,7].

### 2.1. Antidiabetic Significance

**Figure 1:**
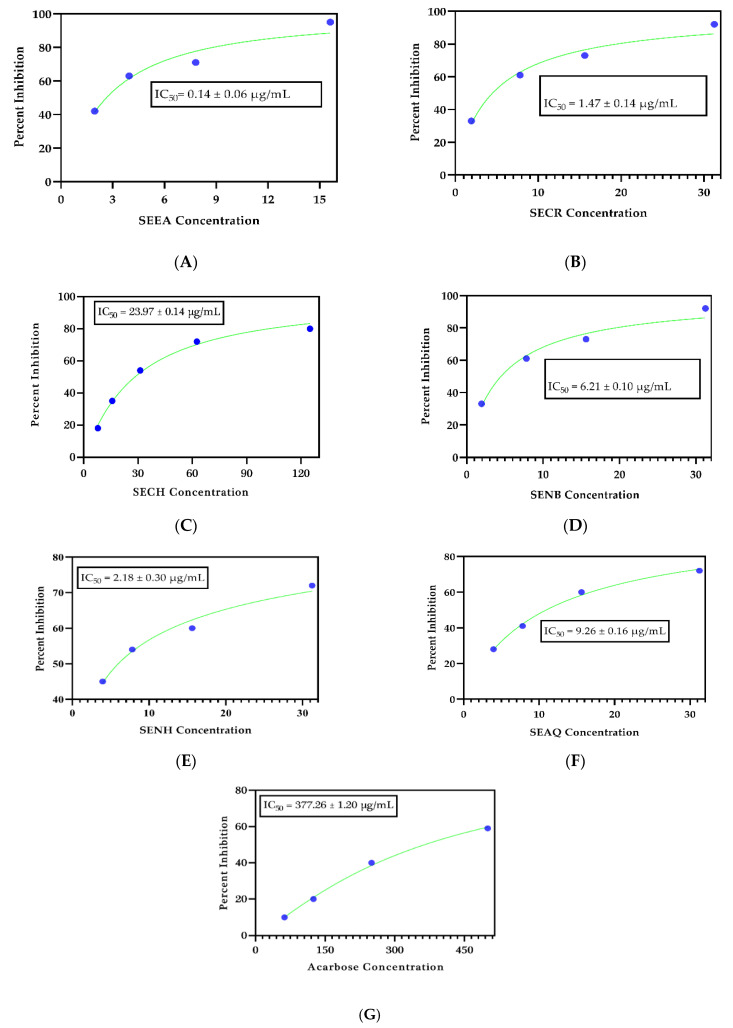
In vitro antidiabetic significance of *S. edelbergii* extract, (**A**) SEEA = *S. edelbergii* ethyl acetate extract, (**B**) SECR = *S. edelbergii* methanolic (crude) extract, (**C**) SECH = *S. edelbergii* chloroform extract, (**D**) SENB = *S. edelbergii* n-butanol extract, (**E**) SENH = *S. edelbergii* n-hexane extract, (**F**) SEAQ = *S. edelbergii* aqueous extract, (**G**) standard = acarbose.

To substantiate the local applications and implications of *S. edelbergii*, the selected plant in the form of crude extract and subfractions was tested in vitro for anti-diabetic ability against yeast α-glucosidase enzymes **(**Figure 1). As we know, diverse medicinal plants and their yields have multiple health benefits [3]. *S. edelbergii* provided insight into their key role in the treatment of DM. The crude extract and subtractions showed potent α-glucosidase inhibitory potential with an IC_50_ in the range of 0.14–23 µg/mL, reflecting a therapeutic approach for the development of new drug candidates and molecules for the treatment of T2DM. The ethyl acetate fraction, followed by crude methanolic, n-hexane and n-butanol, presented appreciable antidiabetic activity with IC_50_ values of 0.14 ± 0.06 µg/mL, 1.47 ± 0.14 µg/mL, 2.18 ± 0.30 µg/mL, and 6.21 ± 0.10 µg/mL, respectively, in comparison with the standard acarbose with IC_50_ = 377.26 ± 1.20 µg/mL, indicating the main anti-diabetic molecules, while the least potential was observed in the aqueous and chloroform fractions with IC_50_ = 9.26 ± 0.16 µg/mL and 23.97 ± 0.14 µg/mL, respectively. Thus, screening showed that our data matched with the findings reported by [8] for *S. baicalensis* antidiabetic assay, and this potential was due to the presence of the active constituents described by [9].

#### ESI-LC-MS Analysis

Based on its significant in vitro antidiabetic potential, the EtOAc fraction was explored for the qualitative detection of the responsible bioactive compounds through ESI-LC-MS profiling using both positive ionization mode (PIM) and negative ionization mode (NIM). The EtOAc fraction presented 16 bioactive compounds corresponding to four major groups: alkaloids, flavonoids, terpenoids, and phenol (Table 1). The ESI-LC-MS full scan chromatograms in PIM and NIM, as well the chromatograms and structures of the identified compounds, are shown in Figures S1 and S2, respectively.

Among the tentatively identified compounds from the EtOAc fraction, the flavonoid was the dominant group, containing eight chemical constituents, six of which **2**, **3**, **4**, **6**, **10**, and **16** were detected in NIM, while two **8** and **13** were observed in PIM. All the flavonoids were previously reported from *S. altissima* [10], *S. baicalensis* [11] *S. amoena* [12], *S. prostrata* [13], *S. amoena* [14], and *S.multicaulis* [15], which have significant antimicrobial, anti-inflammatory, antioxidant and anticancer potential [6] and [16]. These mentioned compounds are reported for the first time from *S. edelbergii*; the EtOAc fraction showed significant ability against various in vitro and in vivo activities, as described by Shah et al. [3]. Moreover, the selected fraction offered five terpenoids, among which **11, 12,** and **15** were tentatively identified in NIM and two—compounds **5** and **9**—were observed in PIM. These compounds from *S. edelbergii* are also explained for the first time, reflecting the literature on *S. rivularis* [17], *S. linearis* [18], *S. albida* [18], and *S. alpina* [19]. The terpenoids were found to have effective anti-cancer, bacteriostasis, anti-virus, and anti-inflammatory properties [20,21]. Two alkaloids—**1** and **7**—were detected in PIM earlier in *S. flavescens* [22] and *S. rivularis* [23] and showed key anti-tumor, anti-stress, and anti-hypoglycemia properties [24]. One phenol (**14**) was also noted in the PIM for its ability to scavenge free radicals and act as an antioxidant and exhibited resistance to human pathogenic bacteria and the ability to serve as an antibacterial agent [25]; it was isolated for the first time from *S.edelbergii* and had been mentioned in the literature on *S. baicalensis* [26].

### 2.2. Computational Analysis

#### 2.2.1. Database of the Compounds Identified in EtOAc Extract

**Figure 2:**
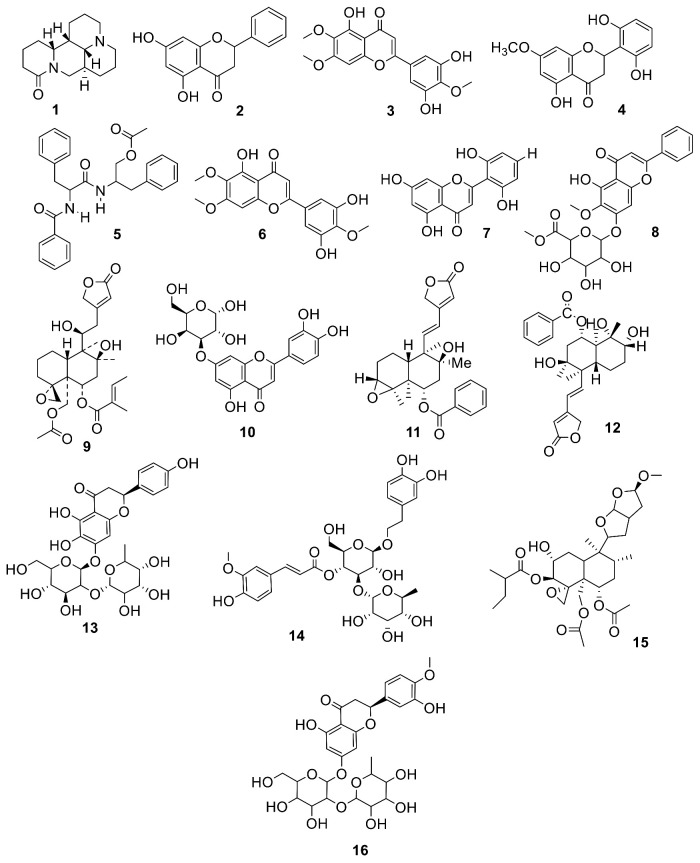
Compounds (**1**–**16**) identified in the EtOAc fraction of *S. edelbergii*.

The compounds identified from the EtOAc fraction of *S.edelbergii* via ESI-LC-MS analysis were used to generate a database with MOE software [27]. Among the tested crude extract and subfractions, the EtOAc fraction presented the maximum potential in vitro as an antidiabetic agent, and on that basis was selected as a significant candidate for further validation using a computational approach; inhibitory constant values reported in studies are included in Table 1. Sixteen compounds were identified from the EtOAc fraction, the chemical structure of which is presented in Figure 2. Each compound was studied for multiple therapeutic options and manages blood glucose levels. Therefore, in this study, computational techniques were used to screen α-glucosidase drugs by computer-aided drug design (CADD) applications, which showed that the drugs could bind significantly within the substrate-binding pocket.

#### 2.2.2. Protein-Ligand Interaction Analysis

**Figure 3:**
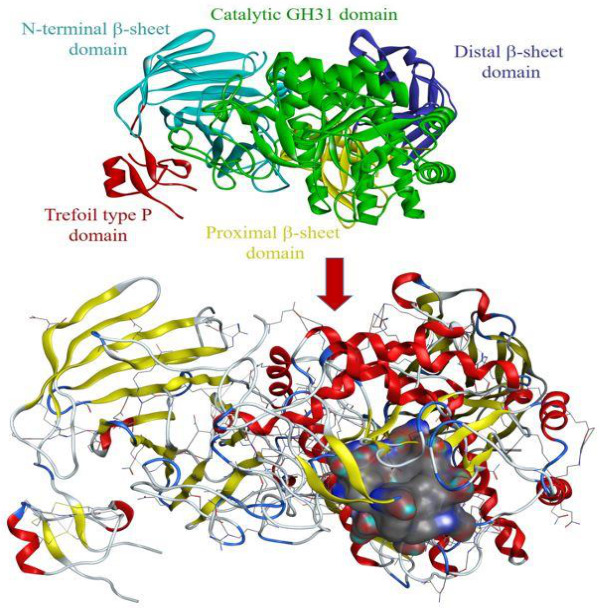
Three-dimensional representation of protein alpha-glucosidase (PDB ID: 5NN5) demonstrating identified functional domains with substrate-binding active sites (structure in surface representation) by Molecular Operating Environment (MOE) software.

The structural information on the α-glucosidase enzyme (PDB ID: 5NN5) was used. The prepared enzyme/protein structure without any bounded ligand and considerable active sites for molecular docking and interaction analysis was identified from literature [28], as shown in Figure 3. A database of 16 bioactive compounds was identified through ESI-LC-MS and subjected to MOE with mdb extension, and protein-ligand docking simulations in the active site of the α-glucosidase enzyme were performed using the Dock module of MOE software [29] (Figure 3). Phytochemicals with the best binding poses and molecular interactions with the significant amino acids elucidated the mechanism of inhibition of α-glucosidase enzyme to manage DM disease. Through evaluation of selected active binding sites, the following residues (TRP376, ASP404, LUE405, ILE441, TRP481, ASP518, MET519, ARG600, TRP613, ASP616, PHE649, and HIS674) were highlighted for significant activity. Previously reported domains and active binding sites of the α-glucosidase enzyme are highlighted in Figure 3 in different colors. The small substrate-binding regions of amino acids located near the C-terminal of beta-strands of the catalytic domain presented as a loop from the region of the N-terminal beta-sheet domain (green and yellow colored representation). Table 2 summarizes the findings of molecular docking analyses of the 16 selected bioactive compounds. The selected compounds demonstrated the best binding poses with a very good dock score within the range of −8.2278 to −5.6735 kcal/mol. All compounds demonstrated a significant dock score along with the number of hydrogen bonds (HBs) within the range of 4.5 Å.

**Figure 4:**
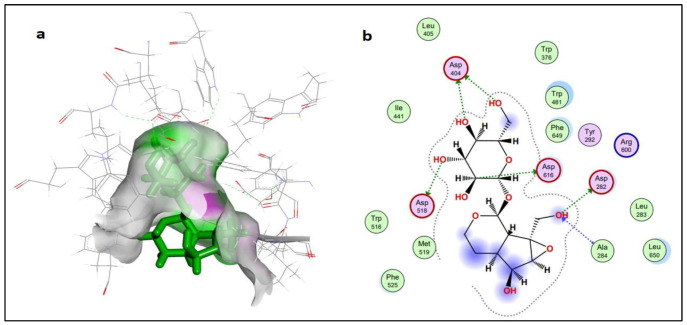
Schematic representation of dihydrocatalpol in the active site of protein alpha-glucosidase (PDB ID 5NN5). The active binding site shows hydrogen bonding capacity as donor atoms (purple) and acceptor atoms (green) (**a**); a two-dimensional plot showing hydrogen bonding interactions and other important hydrophobic interacting residues of the target protein (**b**).

**Figure 5:**
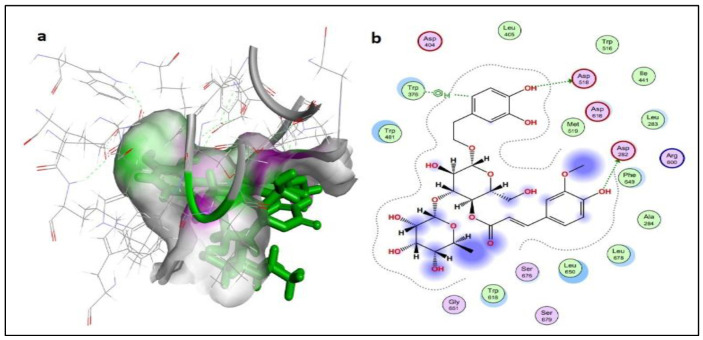
Schematic representation of leucosceptoside A in the active site of protein alpha-glucosidase (PDB ID 5NN5). The active binding site shows hydrogen bonding capacity as donor atoms (purple) and acceptor atoms (green) (**a**); a two-dimensional plot showing hydrogen bonding interactions and other important hydrophobic interacting residues of the target protein (**b**).

Compound **5** (dihydrocatalpol) and compound **14** (leucosceptoside A) presented very good docking findings in terms of binding energy and interactions with key amino acid residues. The threshold value for the acceptable docking score was −6.0 Kcal/mol. Thirteen compounds presented the best pose with a significant docking score above −6.0 kcal/mol. Four compounds (**1**, **6**, **9**, and **15**) did not present interactions with the active site residues of the α-glucosidase protein. However, compound **5** showed the best-bounded conformation at energy value of −6.6079 kcal/mol, and a maximum of six hydrogen bonds were generated with the ASP282(A), ALA284(A), ASP404(A), ASP518(A), and ASP616(A) active residues of target protein (Figure 4). Compound **14** presented more significant binding energy at −7.5862 kcal/mol and generated two hydrogen bonds with ASP282(A) and ASP518(A) residues in the vicinity of the active bonding cleft of the target protein, while one Pi interaction also assisted by TRP376(A) residues, yielding a stable conformation in the docked complex (Figure 5).

#### 2.2.3. Molecular Dynamics (MD) Simulations

**Figure 6:**
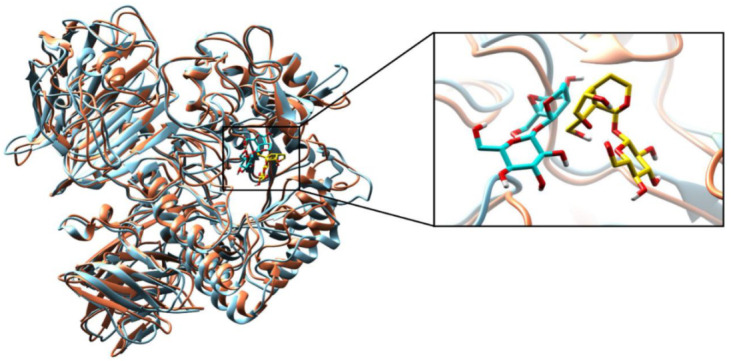
Superimposition of protein alpha-glucosidase complex with dihydrocatalpol at 0 ns and 100 ns. The calculated RMSD value is 1.08 Å; protein at 0 ns is presented in coral and ligand in gold color, while protein at 100 ns is presented in sky blue and ligand in cyan color. Critical structural changes are shown in zoomed view.

**Figure 7:**
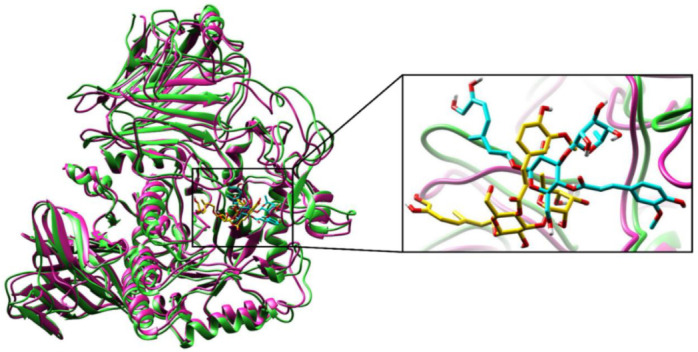
Superimposition of protein alpha-glucosidase complex with leucosceptoside A at 0 ns and 100 ns. The calculated RMSD value is 1.17 Å; protein at 0 ns is presented in violet and ligand in gold color, while protein at 100 ns is presented in lime green color and ligand in cyan color. Critical structural changes are shown in zoomed view.

To validate the structural behavior of selected bioactive compounds within the substrate-binding active cavity of α-glucosidase, MD simulations were performed at 100 ns. α-glucosidase enzyme stability is the description of all the total forces to determine whether the protein will remain in folded state or assume non-native congregating structures. Superimposition of α-glucosidase protein and dihydrocatalpol inhibitor complex at 0 ns and 100 ns is illustrated in Figure 6. Therefore, comparing the protein-ligand complex conformations at a different level of stimulation until 100 ns provided highly useful structural insights that help to understand the possible changes in the ligand pose generated as a result. Hence, an RMSD of 1.08 Å was noted during complex comparison, protein at 0 ns (coral color) and ligand (gold color), while at 100 ns the protein (sky blue color) and ligand (cyan color) showed the stable conformation during simulation; the complex structural comparison indicates minor divergence in the conformation due to little movement in ligand pose from the docked pose, and the correlation of interactions proved that the simulation results confirmed the stability of α-glucosidase protein and dihydrocatalpol inhibitor complex. The superimposition of α-glucosidase and leucosceptoside A inhibitor complex at 0 ns and 100 ns is illustrated in Figure 7. During the critical investigation of structural changes, the RMSD value was calculated as 1.17 Å in the superimposed complex. In graphical representation, the protein at 0 ns is in violet color and ligand is in gold, while protein at 100 ns is lime green in color and ligand is in cyan.

**Figure 8:**
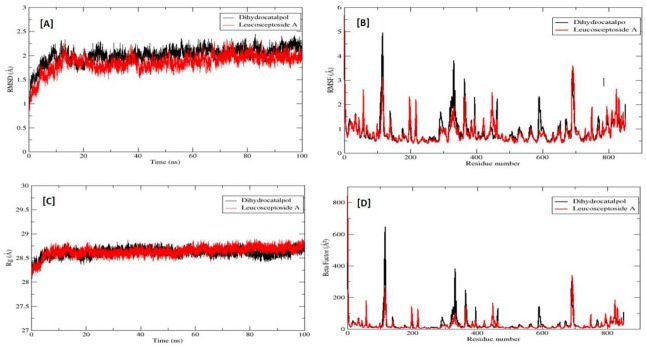
Root mean square deviation (RMSD) of protein and α-glucosidase inhibitor complexes (**A**) in Angstrom (Å) units is plotted on Y-axis, while the variation of bonded conformation through time in nanoseconds (ns) is plotted on the X-axis. Root mean square fluctuation (RMSF) of protein α-glucosidase (**B**) in Angstrom (Å) units is plotted on the Y-axis while the X-axis plots the index of the residue during from 0 ns to 100 ns. The radius of gyration (Rg) of protein and alpha-glucosidase inhibitor complexes (**C**) is plotted on the Y-axis in Angstrom (Å) units, while the X-axis plots the variation of bonded conformations throughout the simulations. A beta factor of protein alpha-glucosidase (**D**) is plotted on the Y-axis in Angstrom (Å) units, while the X-axis plots the index of the residue from 0 ns to 100 ns.

During the simulation, 2D plots were generated to explain the fluctuating behavior of the docked complex at different time frames during MD simulation productions. These plots were important for statistical analysis of the MD simulations; hence, it could be significant to decode the backbone stability and flexibility of the residues during the different time frames of MD simulations [27]. RMSD was calculated as the small atoms convergence from a reference state (Figure 8A), while the RMSF of a protein explained the protein’s dynamic nature that contributed to the system’s overall versatility and residual mobility from its mean position (Figure 8B). The average RMSD value was noted as 0.904 Å, and the average RMSF value was noted as 2.019 Å for the dihydrocatalpol–α-glucosidase complex. However, it is noted that the average RMSD value was 1.863 Å and the average RMSF value was 0.847 Å for leucosceptoside A–α-glucosidase complex; it was observed that key residues were involved in the binding interactions at 0 ns and 100 ns, showing maximum correlation with the docking results. Additionally, minor fluctuations of the α-glucosidase macromolecule residues were observed from the initial state during MD simulation runs during the 0–100 ns simulation period.

The radius of gyration (Rg) was evaluated to confirm the compactness and equilibrium conformation of the system; it is expected that the high and low values of the radius of gyration describe the magnitude of system compactness and a less-tight packing scheme. The estimated average Rg of the system was 28.616 Å with a maximum value of 28.741 Å, reflecting the highest peak of trajectories and compact nature of the system for dihydrocatalpol–α-glucosidase complex; and an average value of 28.644 Å with the maximum value of 28.703 Å, reflecting the highest peak of trajectories for leucosceptoside A–α-glucosidase complex (Figure 8C). The thermal residual deviation was calculated afterward via beta-factor (BF) (Figure 8D) and seemed effectively correlated with RMSF, thus validating the stability of the system. BF and RMSF are complements of each other in terms of overall system stability and residual flexibility. The average BF of the system analyzed for dihydrocatalpol and the enzyme complex was 28.839 Å, and for leucosceptoside A and enzyme complex, it was 24.7687 Å.

**Figure 9:**
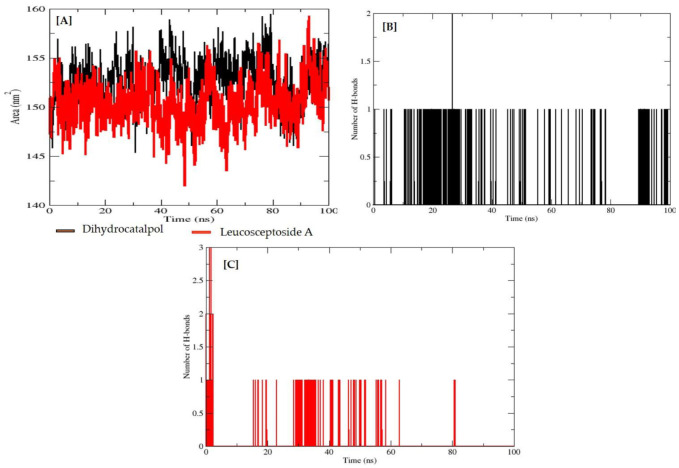
Solvent-accessible surface area (SASA) of α-glucosidase protein (**A**). Y-axis plots the thermodynamic calculations of changes in alpha-glucosidase complex surface area in nanometers squared (nm^2^) (Å) while X-axis plots residue stability time from 0 ns to 100 ns. The number of hydrogen bonds of alpha-glucosidase–dihydrocatalpol complex from 0 ns to 100 ns (**B**) and leucosceptoside A (**C**). Y-axis plots the calculated number of hydrogen bonds while X-axis plots variation in time during molecular dynamic simulations.

Solvent accessible surface area (SASA) is a very expedient examination in which changes in the accessibility of the α-glucosidase to solvent were determined. The stability of each complex was perceived during the simulation, and the average value of SASA was calculated at 151.65 nm^2^ for dihydrocatalpol and 150.41 nm^2^ for leucosceptoside A (Figure 9A).

The frequency of the hydrogen bonds (HBs) plays an important role in overall complex stability; an increase in HBs boosts the binding affinity of the biomolecule for the protein binding site and thus strengthens the complex stability. These hydrogen bonds were obtained by VMD hydrogen bond plugin. During MD analysis from 0–100 ns, the estimated maximum number of HBs between α-glucosidase residues and dihydrocatalpol atom was 2, and the minimum number of HBs was 1 (Figure 9B), while the estimated maximum number of HBs between α-glucosidase residues and leucosceptoside A atom was 2 and the minimum number of HBs was 1 ns (Figure 9C).

#### 2.2.4. Binding Free Energy Calculations (MM-GBSA/ MM-PBSA)

In molecular mechanics (MM), PBSA and GBSA energy model generation are the acceptable and necessary techniques used in the biomolecular investigation of binding free energy calculations of selected protein-ligand complexes and support the analysis of protein folding and stability in drug design and discovery [27]. The MM energy of the selected complex (ΔTOTAL) followed by generalized Born surface area (MM/GBSA) outline the well-organized estimations without any loss in terms of accuracy, and Poisson Boltzmann (MM/PBSA) benchmark parameters could be significant to expose the promising macromolecule α-glucosidase-dihydrocatalpol and -leucosceptoside A ligand complexes in pure water.

The total energy estimated for the MM/GBSA and MM/PBSA models of the dihydrocatalpol complex were −26.2923 and −10.5697 kcal/mol, respectively, while those for the leucosceptoside A complex were −18.3588 and −15.3721 kcal/mol, respectively. For molecular mechanics energy parameters, a strong influence was observed from the gas-phase energy (ΔG gas) when assessed against it, extremely minor contributions from the solvation energy (ΔG solv).

In the MM/GBSA model of the dihydrocatalpol complex, the ΔG gas energy was estimated at −78.0531 kcal/mol; however, in the MM/PBSA model, the same energy parameters estimation was recorded at −78.0531 kcal/mol. In the MM/GBSA model of the leucosceptoside A complex, the ΔG gas energy was estimated at −41.0979 kcal/mol, while in the MM/PBSA model, the ΔG gas energy estimate was the same, at −41.0979 kcal/mol. For the dihydrocatalpol complex, the ΔG solv energy for the MM/GBSA model was projected at 51.7608 kcal/mol, though in the case of MM/PBSA, it was observed to be 67.4834 kcal/mol. For leucosceptoside A complex, the ΔG solv energy in the case of the MM/GBSA model was 22.7391 kcal/mol and for the MM/PBSA model, 25.7258 kcal/mol. For the dihydrocatalpol complex, the electrostatic forces play a role in the stability of the complex. The system estimated by MM forces field in PBSA and GBSA models were in the range of total premeditated energy calculated at −44.2017 kcal/mol for dihydrocatalpol complex and -14.9926 kcal/mol for leucosceptoside A complex. Similarly, the van der Waals forces were also estimated from MM associated with the system stability as −33.8515 kcal/mol for dihydrocatalpol complex and −26.1053 kcal/mol for leucosceptoside A complex. For the dihydrocatalpol complex, an electrostatic energy impact (EGB and EPB) to the ΔG solv was observed, the prime constraint leading towards the out of the range calculations in MM/GBSA solv energy. The surface area energy highlighted as ESURF computed in the MM/GBSA model was −4.6737 kcal/mol. In the MM/PBSA model, the two important terms ENPOLAR and EDISPER, the repulsive and attractive free energies, were presented as −3.9887 and zero kcal/mol. For leucosceptoside A complex, the electrostatic energy impact, EGB and EPB, to the ΔG solv was counted as a non-favorable result in MM/GBSA solv energy. Total ESURF in the MM/GBSA was calculated at −2.9626 kcal/mol, whereas in the MM/PBSA model, ENPOLAR and EDISPER were −2.4337 and zero kcal/mol, correspondingly. The individual total bounded conformations presenting the free energy for the selected α-glucosidase macromolecule as receptor and selected phytochemical complex as ligand (5 and 14) are explained in Table 3.

#### 2.2.5. Pharmacokinetic /ADMET Profile Estimation

Several studies have proved the importance of pharmacokinetic/ADMET profile estimation for the screening of databases to identify potential drug-like and lead-like compounds that could be better tolerated in the design and development of new drugs [29]. ADMET properties were calculated by Swiss ADME [27] and Data warrior tools [29]. Physicochemical properties of the two selected compounds, such as partition coefficient/Lipophilic parameters (log P values), hydrogen bond acceptor (HBA), hydrogen bond donor (HDB), total polar surface area (TPSA), molar refractivity (MR), and rotatable bond (RB) were calculated for each compound. As initial phase drug discovery protocols promote the calculation of drug-likeness, water solubility, pharmacokinetics, and toxicity estimations, these parameters are presented along with the medicinal chemistry perspective for the 10 top-scored compounds in Table 4.

Due to the large chemical structure of natural compounds in the selected dataset, physicochemical properties described by Ismail et al. [27] and Shaker et al. [29] theory of drug-likeness violate one or more parameters shown in Table 4. Compound 4 (dihydrocatalpol) has good bioavailability compared to compound 7 (leucosceptoside A). Toxicity estimates for both compounds presented significant results with minor violations. While dihydrocatalpol presented the expected mutagenic effect, leucosceptoside A presented no predicted toxicity in any of the four major parameters consisting of mutagenicity, tumorigenicity, reproductive effects and irritant effects. The lipophilic profile and water solubility of both the dihydrocatalpol and leucosceptoside A compounds were quite good values. Both phytochemicals presented structural alerts in the medicinal chemistry perspective evaluation, which are important preliminary predictions. Pharmacokinetic properties of both selected compounds varied in other drug-like parameters because most plant-derived compounds do not fulfill the Lipinski rule of drug-likeness due to their large chemical structures [29].

## 3. Material and Methods

**Figure 10:**
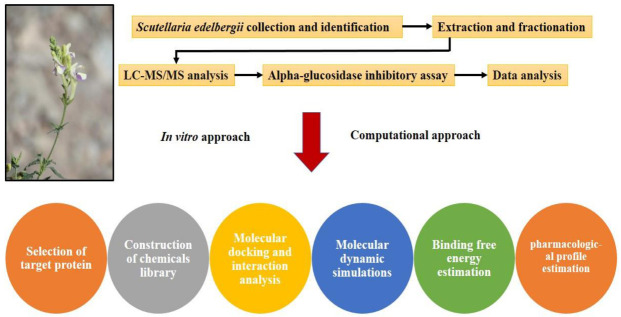
Pipeline for identification and screening of small drug-like potential alpha-glucosidase inhibitors for diabetes mellitus.

The pipeline used for the identification and screening of small drug-like potential α -glucosidase inhibitors of DM explaining the significance of in vitro and computational approaches is shown in Figure 10. Phytocompounds were isolated and subjected to computational investigation to define the mechanism of α -glucosidase inhibition by molecular docking and dynamic simulations.

### 3.1. Apparatus Used

LC-MS/MS (LTQ XL, Thermo Electron Corporation, Waltham, MA, USA) and Xcalibur 2.2 software (Thermo Fisher Scientific, Waltham, MA, USA) were purchased from Fisher Scientific (Illkirch, France). The solvents used for liquid chromatography were LC-MS grade acetonitrile (Fisher Scientific). Deionized water was purified by a Milli-Q (Millipore, Bedford, MA, USA) water purification system.

### 3.2. Plant Collection and Identification

*S. edelbergii* was harvested in the flowering season (April–June 2019) from mountain trails (1660–2200 m) in Kalam, District Swat, Khyber Pakhtunkhwa, Pakistan, and identified by the plant taxonomist Prof. Mehboob Rahman, Matta College Swat, KPK, Pakistan. The plant specimens were deposited at the herbarium (AWKUM/Herb/ 2234), Department of Botany, Abdul Wali Khan University, Mardan, Pakistan [3]. *S. edelbergii* is a perennial herb, propagating through its woody rootstock, with slender stems, procumbent or weakly ascending, round-quadrangular, leaves much-branched, with ovate or acute margins. The flowers are subtended, small calyx with purple scutellum, enlarged during fruit. The petals are yellow or sometimes blue-violet, lower lip darker, spreading erect or erect, densely glandular pilose. Flowering is produced in April to July, found between 1660–2200 m.

### 3.3. Extraction and Fractionation

The *S. edelbergii* specimens were cleaned via tap water to eliminate the unwanted particles and dehydrated at room temperature under shade for 3 weeks. The dried samples were mashed into fine powder with an electric blender. The obtained plant powder was weighed (2 kg) and placed in a refrigerator at 4 °C. The plant powder was subjected to extraction and fractionation using a systematic technique. Two kg of the plant powder was soaked in a glass container (6 L) with commercial-grade methanol, followed by shaking, and then filtered through a muslin cloth after 21 days. The resulting filtrates underwent solvent evaporation via Rota-vapor under controlled temperature (40 °C). The attained semi-solid paste was placed in the open air to dry, yielding a 600-g crude extract that was then kept in a sealed container for further use. To prepare different fractions, 500 g of crude extract was immersed in 1 L distilled water and then subjected to solvent-solvent extraction in a separating funnel from less to more polar solvents (n-hexane < chloroform < EtOAc < n-BuOH). The resulting extracts were vaporized through a rotary evaporator at 40 °C, yielding 21, 19, 20, 18, and 35 g of n-hexane, chloroform, EtOAc, n-BuOH, and aqueous fractions, respectively [3].

### 3.4. α-Glucosidase Inhibitory Assay

The α-glucosidase assay was performed using 50 mM phosphate buffer (pH 6.8). All samples were dissolved in DMSO. An enzyme (2 U/2 mL) 20 µL/well and test samples (0.5 mg/mL) 20 µL/well and 135 µL/well phosphate buffer were loaded into a 96-well plate, followed by 15 min of incubation at 37 °C. Substrate *p*-nitrophenyl-*α*-D-glucopyranoside (0.7 mM) 25 µL/well was added to the 96-well plate after 15 min of incubation, and readings were measured at 400 nm for 30 min. DMSO 7% was used as a positive control. Percent inhibition was calculated by using the following formula [30].

$$\%\;\mathrm{Inhibition}=100\times \frac{O.D\;\mathrm{of}\;\mathrm{tested}\;\mathrm{samples}}{O.D.\;\mathrm{of}\;\mathrm{control}}-100\;$$

OD is the optical density of the tested samples.

### 3.5. LC-MS/MS Analysis

The EtOAc fraction of *S.edelbergii* displayed significant in vitro antidiabetic potential, based on qualitative detection of the chemical constituents in the selected fraction using linear ion trap mass spectrometer coupled with electrospray ionization source using positive and negative ionization mode via standard approach [31]. The (identification) detection was achieved via direct injection mode (DIM) using electron spray ionization (ESI). The capillary voltage was 3.3 kV, with a temperature of 280 °C, whereas the sample flow rate was set up at 10.5 μL/min. The optimized mass range was from 50–2000 *m*/*z*. The collision-induced dissociation (CID) energy ranged from 10–30, and during MS/MS was maintained in the range of 10–45, depending upon the nature of the parent molecular ion. As a mobile phase, the ratio of methanol and acetonitrile was 80:20 (*v*/*v*). The MS parameters for each compound were adjusted to verify the most satisfactory ionization and ion transfer conditions. The best possible signals of both the precursor and fragment ions were attained by infusing the analytes and manually adjusting the parameters. The source parameters were identical for all analytes.

### 3.6. Computational Analysis

#### 3.6.1. Construction of Chemical Database for In Silico Screening

Recent literature highlights the importance of identifying promising drugs for DM because previously reported synthetic drugs have certain unhealthy side effects [28]. Virtual screening techniques are highly recommended to find potential drugs for fatal and infectious diseases [29]. An integrated CADD scheme was employed using 16 bioactive compound databases to identify potential drugs to combat DM. The structure of each compound was drawn using ChemDraw software [32], and information on each structure was crosschecked from the PubChem database [33] to reduce the chance of ambiguity and saved in SDF format for subsequent investigations.

#### 3.6.2. Selection of Target Protein

The selection of an appropriate protein structure to initiate the drug design pipeline defines the important parameters necessary to clarify the action of bound ligands [34], which could selectively inhibit the activity of the α-glucosidase enzyme and hence improve insulin expression in DM patients [35]. Therefore, α-glucosidase protein (PDB ID:5NN5) was selected to perform a molecular docking experiment in this study to evaluate the protein–ligand binding interactions [36].

#### 3.6.3. Protein-Ligand Docking and Interactions Analysis

Molecular docking is an important CADD application for the identification of protein-ligand interactions to understand the molecular mechanism of small drug-like entities in cellular pathways [28]. ESI-LC-MS identified 16 compounds that were used for molecular docking, and the 3D structure of the α-glucosidase protein (PBD ID: 5NN5) in pdb format was imported to the MOE software [37]. Heteroatoms, 3D protonation, and water molecules along with the default ligand attached to the target protein were removed to prepare the protein for the docking procedure shown in Figure 3. An active site was identified in the selected protein (5NN5) based on previous literature [36], and structural optimization was performed using steps such as the addition of hydrogen atoms and energy minimization with the Amber14 force field method applied with chiral constraints and geometrical parameters. By using the surfaces and maps panel module, transparency of the front and back surfaces was adjusted, resulting in information on significant residues in the selected substrate binding site of 5NN5 protein in the native conformation [36]. MOE software created a database of 16 compounds identified from experimental studies to perform molecular docking simulations and saved it with the mdb extension for further analysis. Top-ranked poses were subjected to refinement and calculation of binding free energies (ΔG), which were evaluated by the scoring function GBVI/WSA dg [38]. A reliable scoring scheme that resulted in the docking score of the correct binding poses was established by the number of molecular interactions (hydrogen, Pi, and hydrophobic interactions) [39]. The MOE database of the docked complex was carefully visualized to understand the mode of binding interactions of α-glucosidase inhibitors bound in the selected pocket of the target protein.

#### 3.6.4. Molecular Dynamics Simulations

The two selected lead compound complexes were used for further MD simulation analysis at 100 ns to understand the behaviors of the docked complex and conformational stability. MD simulation steps were (1) preparation (2) pre-processing and (3) simulation run [40]. Amber 20 software was used for the MD simulations. Initial system preparation was performed with the antechamber module [41], followed by complex library adjustments, and the parameters were fixed for α-glucosidase protein and selected ligands. In the solvation system, the complex was solvated (12 Å) with the Leap module of the AMBER 20 software. Macromolecule binding was resolved by ff14SB force field [42] and charge neutralization, Na^+^ ions were also processed [43].

Pre-processing fixed the selected complex energy calculations and completed the set of hydrogen bonds (HBs), which were intended for 500 steps, minimization of solvation system energy for 1000 steps, limited to 200 kcal/mol-Å^2^ on the residual system; minimization of the complete set of system atoms was performed once more for 1000 steps with pragmatic restraint of 5 kcal/mol-Å^2^ applied to system carbon α atoms and 300 steps of minimization on system non-heavy atoms with the restraint of 100 kcal/mol-Å^2^ on other system modules. The protein-ligand complexes were heated progressively to 300 K by NVT ensemble and preserved by the Langevin dynamics [44] and SHAKE algorithm [45] to confine the HBs. The equilibration stage of the protein-ligand complexes was recovered at 100 ps. The pressure was applied in the MD simulation system using NPT ensemble and restraining the system to Cα atoms of 5 kcal/mol-Å^2^. The MD simulation plots of 0 ns to 100ns were generated using a time scale of 2 fs.

Before and after binding, significant interaction estimations were performed by maintaining a cut-off range of 8.0 Å. The CPPTRAJ module was applied for statistical estimations to ensure structural stability in the best conformation [46]. The MD simulation plots were generated by Visual Molecular Dynamics (VMD) software [47].

#### 3.6.5. Binding Free Energy (BFE) Estimation

Amber 20 was used for the estimation of binding interactions and solvation free energies for the α-glucosidase protein target. Docked protein-ligand complex was subjected to MM-PBSA calculation for absolute binding free energy estimation, which was the sum of gas-phase and solvation free energies during MD simulation for 100 ns [48]; corresponding MM-GBSA calculations were performed to identify differences between the bound and unbound presentations of solvated conformations of the selected potential target [47]. Mathematically, the BFE can be analyzed by the following Equation (1).

(1) $$\Delta G_{\;\mathrm{bind},\mathrm{solv}}=\Delta G_{\;\mathrm{bind},\;\mathrm{vacuum}}+\Delta G_{\;\mathrm{solv}},\;\mathrm{protien}-\mathrm{ligand}-(\Delta {G\;}_{\mathrm{solv}},\;\mathrm{ligand}+\Delta G_{\;\mathrm{solv},\;}\;\mathrm{protein}$$

For all three conditions of the MM system, the solvation energy for the transfer of molecules from the gas phase to solvent was assessed by resolving any one Poisson-Boltzmann (PB) or generalized Born (GB) equation. Therefore, it contributed to the electrostatic role of the solvation phase. Similarly, it allowed the calculation of empirical terms for hydrophobic forces as presented in Equation (2).

(2) $$\Delta G_{\;\mathrm{solv}}=G_{\;\mathrm{electrostatic},\;\epsilon =80}-G_{\;\mathrm{electrostatic},\;\epsilon =1}+\Delta G_{\;\mathrm{hydrophobic}\;})\;$$

The estimation of the average interaction energy between the ligand and protein yields ΔG _vacuum_ (Equation (3)).

(3) $$\Delta G_{\;\mathrm{vacuum}}=\Delta E_{\;\mathrm{molecular}\;\mathrm{mechanics}}-T.\Delta S$$

#### 3.6.6. Pharmacokinetic /ADMET Profile Estimation

Based on docking results, the best bioactive compounds were used for the calculation of the ADMET (absorption, distribution, metabolism, excretion, and toxicity) profile, which is an essential criterion for the screening of drug-like chemical compounds [49,50]. For ADMET profile estimation of selected phytochemicals, Swiss ADME [34], Data Warrior [51], and computational tools [52] were used.

### 3.7. Data Analysis

The compounds detected through ESI-LC-MS screening were identified using online database software [47,48] with already-reported literature on the genus *Scutellaria* cited against each compound; further, Xcalibur 2.0.7 (software) was applied for data analysis and acquisition.

## 4. Conclusions

Medicinal plants have provided numerous therapies due to the presence of bioactive compounds. The current study highlighted the importance of *S. edelbergii* crude extract and subfractions against the antidiabetic assay, demonstrating significant potential in general, whereas the EtOAc fraction was the most effective. The active fraction was profiled for the identification of biopotent compounds, yielding 16 bioactive constituents. Their significance was further validated through computational analysis. Molecular docking investigations of the 16 compounds led to the identification of two potential hits (dihydrocatalpol and leucosceptoside A). Dihydrocatalpol, a very important anti-inflammatory medicinal agent found in several traditional medicines, presented the best results in terms of energy value (−6.6079 kcal/mol), and generated six hydrogen bonds with the ASP282 (A), ALA284 (A), ASP404 (A), ASP518 (A), and ASP616 (A) residues of the target protein. While leucosceptoside A has benefits in traditional medicine for hyperglycemia and chronic diseases, it presented more significant binding energy (−7.5862 kcal/mol) and generated two hydrogen bonds with ASP282 (A) and ASP518 (A) residues in the vicinity of the active bonding cleft of the target protein, as well as one Pi interaction assisted by TRP376(A) residues, achieving a stable conformation in docked complex with a mechanism of α-glucosidase inhibition that assists the improvement of insulin secretion and manages blood glucose in diabetic patients. Furthermore, MD simulations allowed observation of the stable conformational parameters presenting estimated binding free energies and important trajectories explaining major features of the bounded complex. Very minor fluctuations were observed in terms of RMSD and RMSF values that correlated with the results of molecular docking. Moreover, an additional parameter was applied for ADMET estimations that offered insight into the drug-like characteristics of the two selected potential hits. Hence, these results are highly recommended for further clinical investigations and could facilitate the drug development phase for therapeutic agents against diabetes mellitus.

## Figures and Tables

**Table 1:** Identification of chemical constituents in the EtOAc fraction of *S. edelbergii* via ESI-LC-MS.

No.	RT (Min)	MW IM	Fragmentation	Tentative Identification	Reference Species	Class
**1**	4.87	[249]^+^	240.92, 234.17	Isomatrine	*S. flavescens* [22]	Alkaloid
**2**	5.15	[255]^^−^^	245.25, 237.17	Pinocembrin	*S. altissima* [10]	Flavonoid
**3**	5.35	[285]^−^	270.08, 267.08	5,7,2,6-Tetrahydroxy-flavone	*S. baicalensis* [11]	Flavonoid
**4**	5.50	[301]^−^	286.08, 284.0	Scuteamoenin	*S. amoena* [12]	Flavonoid
**5**	5.74	[365]^+^	350.08, 347.25	Dihydrocatalpol	*S. albida* [18]	Terpenoid
**6**	6.08	[359]^−^	344.08, 315.17	5,2,5-Trihydroxy-6,7,8-trimethoxyflavone	*S. baicalensis* [11]	Flavonoid
**7**	6.43	[445]^+^	430.17, 427.25	Aurantiamide acetate	*S. rivularis* [23]	Alkaloid
**8**	6.53	[475]^+^	460.25, 457.17	OroxylinA-7-*O*-d-glucuronopyranosidemethylester	*S. amoena* [14]	Flavonoid
**9**	6.97	[507]^+^	489.17, 475.25	Scutalpin C	*S. alpina* [19]	Terpenoid
**10**	7.04	[447]^−^	429.17, 403.17	Luteloin-7-*O*-d-glucuronopyranoside	*S. prostrata* [13]	Flavonoid
**11**	7.17	[451]^−^	433.25, 407.25	Scutellone F	*S. rivularis* [17]	Terpenoid
**12**	7.31	[469]^−^	451.17, 425.17	Scutellone D	*S. rivularis* [17]	Terpenoid
**13**	7.43	[595]^+^	565.25, 523.42	Scutellarein-7-*O*-neohesperidoside	*S. multicaulis* [15]	Flavonoid
**14**	7.72	[639]^+^	621.42, 567.42	Leucosceptoside A	*S. baicalensis* [26]	Phenol
**15**	8.02	[581]^−^	545.08, 519.25	Lupulin A	*S. linearis* [18]	Terpenoid
**16**	8.24	[609]^−^	577.33, 565.08	Hesperetin-7-*O*-neohesperidoside	*S. multicaulis* [15]	Flavonoid

IM = ionization mode, MW = Molecular weight, Rt = Retention time.

**Table 2:** Summary of results of molecular docking analysis of 16 selected compounds.

No.	Dock Score	RMSD	Interacting Residues in the Binding Pocket				
			Ligand	Receptor	Interaction	Distance	E (kcal/mol)
**1**	−5.7671	0.81	-	-	-	-	-
**2**	−5.6933	0.97	C6 O12	SD:MET519(A) OD1:ASP404(A)	H-donor H-donor	3.76 3.06	−0.5 −3.0
**3**	−5.6735	2.40	O20	OD1:ASP518(A)	H-donor	2.93	−3.2
**4**	−6.1364	1.22	O20	OD1:ASP518(A)	H-donor	2.87	−2.1
**5**	−6.6079	1.09	O14 O20 O21 O22 O28 O28	OD2:ASP616(A) OD1:ASP518(A) OD1:ASP404(A) OD1:ASP404(A) OD1:ASP282(A) N:ALA284(A)	H-donor H-donor H-donor H-donor H-donor H-acceptor	3.15 2.99 3.03 3.00 2.93 3.12	−0.5 −1.1 −0.9 −3.0 −0.9 −0.7
**6**	−6.523	1.64	-	-	-	-	-
**7**	−7.1585	1.63	O27	N:ALA284(A)	H-acceptor	3.17	−0.8
**8**	−7.1405	1.09	C17 O19	OD1:ASP282(A) N:ALA284(A)	H-donor H-acceptor	3.28 3.04	−0.7 −1.6
**9**	−7.0130	1.95	-	-	-	-	-
**10**	−6.9602	1.15	O20 O30 O19	O:ASN524(A) OD2:ASP616(A) N:ALA284(A)	H-donor H-donor H-acceptor	3.00 2.97 3.23	−2.7 −2.3 −1.0
**11**	−6.8364	1.60	O20	OD1:ASP282(A)	H-donor	3.02	−1.9
**12**	−6.4003	1.95	O33	NH1:ARG600(A)	H-acceptor	3.16	−1.4
**13**	−8.2278	2.17	O38	OD2:ASP282(A)	H-donor	3.35	−0.5
**14**	−7.5862	2.08	O29 O45 C28	OD1:ASP518(A) OD1:ASP282(A) 6ring:TRP376(A)	H-donor H-donor H-pi	3.32 2.78 4.54	−0.5 −3.4 −0.6
**15**	−77456	2.33	-	-	-	-	-
**16**	−8.169	2.96	O35	OD1:ASP518(A)	H-donor	2.17	−1.2

**Table 3:** Binding free energies of the α-glucosidase protein and selected potential bioactive compound complexes.

C. No	MM/GBSA Model				MM/PBSA Model			
**5**	VDWAALS	−33.8515	2.3527	0.2353	VDWAALS	−33.8515	2.3527	0.2353
	EEL	−44.2017	85.0494	0.5049	EEL	−44.2017	5.0494	0.5049
	EGB	−56.4345	4.7492	0.4749	EPB	71.4721	6.3198	0.6321
	ESURF	−4.6737	0.3065	0.0306	ENPOLAR	−3.9887	0.1867	0.0187
					EDISPER	0	0	0
	ΔG gas	−78.0531	5.3641	0.5364	ΔG gas	−78.0531	5.3641	0.5364
	ΔG solv	51.7608	4.6677	0.4668	ΔG solv	67.4834	6.2590	0.6259
	ΔTOTAL	−26.2923	2.3706	0.2371	ΔTOTAL	−10.5697	4.2572	0.4257
**14**	VDWAALS	−26.1053	1.7151	0.1715	VDWAALS	−26.1053	1.7151	0.1715
	EEL	−14.9926	4.3080	0.4308	EEL	−14.9926	4.3080	0.4308
	EGB	25.7016	4.7242	0.4724	EPB	28.1595	5.1235	0.5123
	ESURF	−2.9626	0.1831	0.0183	ENPOLAR	−2.4337	0.0970	0.0097
					EDISPER	0	0	0
	ΔG gas	−41.0979	5.3198	0.5320	ΔG gas	−41.0979	5.3198	0.5320
	ΔG solv	22.7391	4.6019	0.4602	ΔG solv	25.7258	5.0694	0.5069
	ΔTOTAL	−18.3588	1.6221	0.1622	ΔTOTAL	−15.3721	2.0689	0.2069

Where **5** = dihydrocatalpol, **14** = leucosceptoside A.

**Table 4:** Summary of drug-likeness and in silico pharmacokinetic properties of the two selected α-glucosidase inhibitors calculated by SwissADME server and Data-Warrior software.

Chemical Parameters	Dihydrocatalpol	Leucosceptoside A
**Physicochemical Properties**		
Molecular weight (MW) (g/mol)	364.35	638.61
Rotatable bonds	4	12
Hydrogen bond acceptors (HBA)	10	15
Hydrogen bond donors (HBD)	6	8
Molar Refractivity (MR)	77.11	152.89
Total polar surface area (TPSA) (Å)	161.60	234.29
Bioavailability Score	0.55	0.17
**Lipophilicity**		
Log Po/w (iLOGP)	1.38	2.78
LogPo/w (XLOGP3)	−3.34	−0.18
LogPo/w (WLOGP)	−3.71	−0.82
Lpg Po/w (MLOGP)	−2.81	−2.18
LogPo/w (SILICOS-IT)	−2.50	−0.58
Consensus Log Po/w	−2.20	−0.20
**Water Solubility**		
Class	Highly soluble	Soluble
**Pharmacokinetics**		
GI absorption	Low	Low
BBB permeant	No	No
P-gp substrate	Yes	Yes
CYP1A2 inhibitor	No	No
CYP2C19 Inhibitor	No	No
CYP2C9 inhibitor	No	No
CYP2D6 inhibitor	No	No
CYP3A4 inhibitor	No	No
Log Kp (skin permeation) (cm/s)	−10.89	−10.32
**Toxicity estimation**		
Mutagenicity	Toxic effects	No toxic effects
Tumorigenicity	No toxic effects	No toxic effects
Reproductive effects	No toxic effects	No toxic effects
Irritant effects	No toxic effects	No toxic effects
**Medicinal chemistry-related properties**		
PAINS	No alert	I alert Catechol_A
Brenk	1 alert Three_membered_ heterocycle	2 alert Catechol, Micheal_acceptor_1
Synthetic accessibility	5.39	6.47

## Data Availability

The data are available to researchers upon request.

## Supplementary Materials

The related data are available online as Figure S1: Full-scan ESI-LCMS chromatogram of the EtOAc fraction of *S.edelbergii*. (A) Negative ionization mode and (B) positive ionization mode, Figure S2: Chromatograms of ESI-LCMS identified compounds in the EtOAc fraction of *S.edelbergii*.

## Sample Availability

Samples of the compounds are not available from the authors.

## References

[1] Bruno M., Piozzi F., Rosselli S. (2002). Natural and hemisynthetic neoclerodane diterpenoids from *Scutellaria* and their antifeedant activity. Nat. Prod. Rep..

[2] Shang X., He X., He X., Li M., Zhang R., Fan P., Zhang Q., Jia Z. (2010). The genus *Scutellaria* an ethnopharmacological and phytochemical review. J. Ethnopharmacol..

[3] Shah M., Murad W., Ur Rehman N., Halim S.A., Ahmed M., Rehman H., Zahoor M., Mubin S., Khan A., Nassan M.A. (2021). Biomedical applications of *Scutellaria edelbergii* Rech. f.: In vitro and in vivo approach. Molecules.

[4] Majid A., Ahmad H., Saqib Z., Ali H. (2015). Potential distribution of endemic *Scutellaria chamaedrifolia;* Geographic Information System and statistical model approach. Pak. J. Bot..

[5] Asgarpanah J., Kazemivash N. (2013). Phytochemistry, pharmacology and medicinal properties of *Carthamus tinctorius* L.. Chin. J. Integr. Med..

[6] Rasul A., Millimouno F.M., Ali Eltayb W., Ali M., Li J., Li X. (2013). Pinocembrin: A novel natural compound with versatile pharmacological and biological activities. BioMed Res. Int..

[7] Krishna P.M., KNV R., Banji D. (2012). A review on phytochemical, ethnomedical and pharmacological studies on genus *Sophora*, Fabaceae. Rev. Bras. Farmacogn..

[8] Waisundara V.Y., Hsu A., Huang D., Tan B.K.-H. (2008). *Scutellaria baicalensis* enhances the anti-diabetic activity of metformin in streptozotocin-induced diabetic Wistar rats. Am. J. Chinese Med..

[9] Salehi B., Ata A., V Anil Kumar N., Sharopov F., Ramírez-Alarcón K., Ruiz-Ortega A., Abdulmajid Ayatollahi S., Valere Tsouh Fokou P., Kobarfard F., Amiruddin Zakaria Z. (2019). Antidiabetic potential of medicinal plants and their active components. Biomolecules.

[10] Tomimori T., Miyaichi Y., Imoto Y., Kizu H., Namba T. (1986). Studies on the nepese crude drugs. VI.: On the flavonoid constituents of the root of *Scutellaria discolor* Colebr.(2). Chem. Pharm. Bull..

[11] De Smet P. (1993). Scutellaria Species. Adverse Effects of Herbal Drugs 2.

[12] Hu B., Liu Y. (1989). Studies on the structures of new flavonoids from the root of *Scutellaria amoena*. Acta pharm. Sin..

[13] Kikuchi Y., Miyaichi Y., Yamaguchi Y., Kizu H., Tomimori T. (1991). Studies on the nepalese crude drugs. XII. On the phenolic compounds from the root of *Scutellaria prostrata* Jacq. ex Benth. Chem. Pharm. Bull..

[14] Zhou Z.-H., Zhang Y.-J., Yang C.-R. (1999). New flavonoid glycosides from *Scutellaria amoena*. Plant Sci. J..

[15] Dehkordi F.J., Kharazian N., Lorigooini Z. (2020). Characterization of flavonoid components in *Scutellaria* L. species (Lamiaceae) using finger-printing analysis. Acta Biol. Crac. Ser. Bot..

[16] Rashid M., Fareed M., Rashid H., Aziz H., Ehsan N., Khalid S., Ghaffar I., Ali R., Gul A., Hakeem K.R. (2019). Flavonoids, and their biological secrets. Plant and Hum. Health.

[17] Lin Y.-L., Kuo Y.-H., Cheng M.-C., Wang Y. (1988). Structures of scutellones D and E determined from X-ray diffraction, spectral and chemical evidence. Neoclerodane-type diterpenoids from *Scutellaria rivularis* WALL. Chem. Pharm. Bull..

[18] Hussain H., Ahmad V.U., Anwar S., Miana G.A., Krohn K. (2008). Chemical constituents of *Scutellaria linearis*. Biochem. Syst. Ecol..

[19] Muñoz D.M., Maria C., Rodríguez B., Simmonds M.S., Blaney W.M. (1997). Neo-clerodane insect antifeedants from *Scutellaria alpina* subsp. javalambrensis. Phytochemistry.

[20] Chen Q., Rahman K., Wang S.-J., Zhou S., Zhang H. (2020). *Scutellaria barbata*: A review on chemical constituents, pharmacological activities, and clinical applications. Curr. Pharm. Des..

[21] Denaro M., Smeriglio A., Trombetta D. (2021). Antioxidant and anti-inflammatory activity of citrus flavanones mix and its stability after in vitro simulated digestion. Antioxidant.

[22] Ma S.-C., Du J., But P.P.-H., Deng X.-L., Zhang Y.-W., Ooi V.E.-C., Xu H.-X., Lee S.H.-S., Lee S.F. (2002). Antiviral Chinese medicinal herbs against respiratory syncytial virus. J. Ethnopharmacol..

[23] Lin Y.-L. (1987). Aurantiamide from the aerial parts of *Scutellaria rivularis*. Planta Med..

[24] Lim D.W., Kim Y.T. (2013). Dried root of *Rehmannia glutinosa* prevents bone loss in ovariectomized rats. Molecules.

[25] Brito A., Ramirez J.E., Areche C., Sepúlveda B., Simirgiotis M.J. (2014). HPLC-UV-MS profiles of phenolic compounds and antioxidant activity of fruits from three citrus species consumed in Northern Chile. Molecules.

[26] Zhou Y., Hirotani M., Yoshikawa T., Furuya T. (1997). Flavonoids and phenylethanoids from hairy root cultures of *Scutellaria baicalensis*. Phytochemistry.

[27] Ismail S., Ahmad S., Azam S.S. (2020). Immunoinformatics characterization of SARS-CoV-2 spike glycoprotein for prioritization of epitope based multivalent peptide vaccine. J. Mol. Liq..

[28] Bibi S., Sakata K. (2016). Current status of computer-aided drug design for type 2 diabetes. Curr. Comp. Aided Drug Des..

[29] Shaker B., Ahmad S., Thai T.D., Eyun S.-i., Na D. (2020). Rational drug design for *Pseudomonas aeruginosa* PqsA enzyme: An in silico guided study to block biofilm formation. Front. Mol. Biosci..

[30] Kausar N., Ullah S., Khan M.A., Zafar H., Choudhary M.I., Yousuf S. (2021). Celebrex derivatives: Synthesis, α-glucosidase inhibition, crystal structures and molecular docking studies. Bioorg. Chem..

[31] Khan I., Rahman H., Abd El-Salam N.M., Tawab A., Hussain A., Khan T.A., Khan U.A., Qasim M., Adnan M., Azizullah A. (2017). *Punica granatum* peel extracts: HPLC fractionation and LC-MS analysis to quest compounds having activity against multidrug resistant bacteria. BMC Complement. Altern. Med..

[32] De Sousa Luisa J.A., Barrosb R.P.C., de Sousab N.F., Muratovc E., Scottib L., Scottib M.T. (2021). Virtual screening of natural products database. Mini Rev. Med. Chem..

[33] Kim S., Thiessen P.A., Bolton E.E., Chen J., Fu G., Gindulyte A., Han L., He J., He S., Shoemaker B.A. (2016). PubChem substance and compound databases. Nucleic Acids Res..

[34] Daina A., Michielin O., Zoete V. (2014). iLOGP: A simple, robust, and efficient description of n-octanol/water partition coefficient for drug design using the GB/SA approach. J. Chem. Inf. Model..

[35] Dirir A.M., Daou M., Yousef A.F., Yousef L.F. (2021). A review of alpha-glucosidase inhibitors from plants as potential candidates for the treatment of type-2 diabetes. Phytochem. Rev..

[36] Roig-Zamboni V., Cobucci-Ponzano B., Iacono R., Ferrara M.C., Germany S., Bourne Y., Parenti G., Moracci M., Sulzenbacher G. (2017). Structure of human lysosomal acid α-glucosidase–a guide for the treatment of Pompe disease. Nat. Comm..

[37] Vilar S., Cozza G., Moro S. (2008). Medicinal chemistry, and the molecular operating environment (MOE): Application of QSAR and molecular docking to drug discovery. Curr. Top. Med. Chem..

[38] Yousafi Q., Batool J., Khan M.S., Perveen T., Sajid M.W., Hussain A., Mehmood A., Saleem S. (2021). In Silico Evaluation of Food Derived Bioactive Peptides as Inhibitors of Angiotensin Converting Enzyme (ACE). Int. J. Pept. Res. Ther..

[39] Aldeghi M., Heifetz A., Bodkin M.J., Knapp S., Biggin P.C. (2016). Accurate calculation of the absolute free energy of binding for drug molecules. Chem. Sci..

[40] Ahmad S., Ranaghan K.E., Azam S. (2019). Combating tigecycline resistant *Acinetobacter baumannii*: A leap forward towards multi-epitope-based vaccine discovery. Eur. J. Pharm. Sci..

[41] Lee T.-S., Allen B.K., Giese T.J., Guo Z., Li P., Lin C., McGee Jr T.D., Pearlman D.A., Radak B.K., Tao Y. (2020). Alchemical binding free energy calculations in AMBER20: Advances and best practices for drug discovery. J. Chem. Inf. Model..

[42] Bergonzo C., Cheatham III T.E. (2015). Improved force field parameters lead to a better description of RNA structure. J. Chem. Theory Comput..

[43] Hyun T.K., Eom S.H., Kim J.-S. (2014). Molecular docking studies for discovery of plant-derived a-glucosidase inhibitors. Plant Omics.

[44] Izaguirre J.A., Catarello D.P., Wozniak J.M., Skeel R.D. (2001). Langevin stabilization of molecular dynamics. J. Chem. Phys..

[45] Kräutler V., Van Gunsteren W.F., Hünenberger P.H. (2001). A fast SHAKE algorithm to solve distance constraint equations for small molecules in molecular dynamics simulations. J. Comput. Chem..

[46] Roe D.R., Cheatham III T.E. (2013). Ptraj and Cpptraj: Software for processing and analysis of molecular dynamics trajectory data. J. Chem. Theory Comput..

[47] Humphrey W., Dalke A., Schulten K. (1996). VMD: Visual molecular dynamics. J. Mol. Grap..

[48] Miller III B.R., McGee Jr T.D., Swails J.M., Homeyer N., Gohlke H., Roitberg A.E. (2012). MMPBSA. py: An efficient program for end-state free energy calculations. J. Chem. Theory Comput..

[49] Khan M.S., Mehmood B., Yousafi Q., Bibi S., Fazal S., Saleem S., Sajid M.W., Ihsan A., Azhar M., Kamal M.A. (2021). Molecular Docking Studies Reveal Rhein from rhubarb (*Rheum rhabarbarum*) as a Putative Inhibitor of ATP-binding Cassette Super-family G member 2. Med. Chem..

[50] Avdović E.H., Petrović I.P., Stevanović M.J., Saso L., Dimitrić Marković J.M., Filipović N.D., Živić M.Ž., Cvetić Antić T.N., Žižić M.V., Todorović N.V. (2021). Synthesis and Biological Screening of New 4-Hydroxycoumarin Derivatives and Their Palladium (II) Complexes. Oxid. Med. Cell. Longev..

[51] Sander T., Freyss J., von Korff M., Rufener C. (2015). Data Warrior: An open-source program for chemistry aware data visualization and analysis. J. Chem. Inf. Model..

[52] Avdović E.H., Milanović Ž.B., Molčanov K., Roca S., Vikić-Topić D., Mrkalić E.M., Jelić R.M., Marković Z.S. (2022). Synthesis, characterization and investigating the binding mechanism of novel coumarin derivatives with human serum albumin: Spectroscopic and computational approach. J. Mol. Struct..

