# Supplementary material for: Identification of α-Glucosidase Inhibitors from Scutellaria edelbergii: ESI-LC-MS and Computational Approach
Source: Molecules. 2022 Feb 16;27(4):1322. doi: 10.3390/molecules27041322 (PMC8879825; doi:10.3390/molecules27041322)
Supplement: Supplementary file 1 [file molecules-27-01322-s001.zip › molecules-1521575-supplementary.pdf]

Supporting Information

Identification of α-Glucosidase Inhibitors from *Scutellaria edelbergii*: ESI-LCMS and computational approach

Muddaser Shah[1,2], Hazir Rahman[3], Ajmal Khan[2], Shabana Bibi[4,5], Obaid Ullah[2,6], Saeed Ullah[2,7], Najeeb Ur Rehman[2*], Waheed Murad[1*], and Ahmed Al-Harrasi[2*]

1 Department of Botany, Abdul Wali Khan University Mardan, Mardan 23200, Pakistan; muddasershah@awkum.edu.pk (M.S)

2 Natural and Medical Sciences Research Center, University of Nizwa, P.O. Box 33, Birkat Al Mauz, Nizwa 616, Oman; ajmalkhan@unizwa.edu.om (A.K); aharrasi@unizwa.edu.om (A.A-.H); najeeb@unizwa.edu.om (N.U.R)

3 Department of Microbiology, Abdul Wali Khan University Mardan, Mardan 23200, Pakistan; hazirrahman@awkum.edu.pk

4 Yunnan Herbal Laboratory, College of Ecology and Environmental Sciences, Yunnan University, Kunming 650091, Yunnan, China

5 International Joint Research Center for Sustainable Utilization of Cordyceps Bioresources in China and Southeast Asia, Yunnan University, Kunming 650091, Yunnan, China; shabana_bibi@ynu.edu.cn (S.B).

6 Department of Chemistry, University of Malakand, Chakdara 18800, Pakistan; obaidullah@unizwa.edu.om (O.U)

7 H.E.J. Research Institute of Chemistry, International Center for Chemical and Biological Science, University of Karachi, Karachi-75270, Pakistan. saeedullah@iccs.edu (S.U).

Correspondence: waheedmurad@awkum.edu.pk (W.M); najeeb@unizwa.edu.om (N.U.R); aharrasi@unizwa.edu.om (A.A.-H).

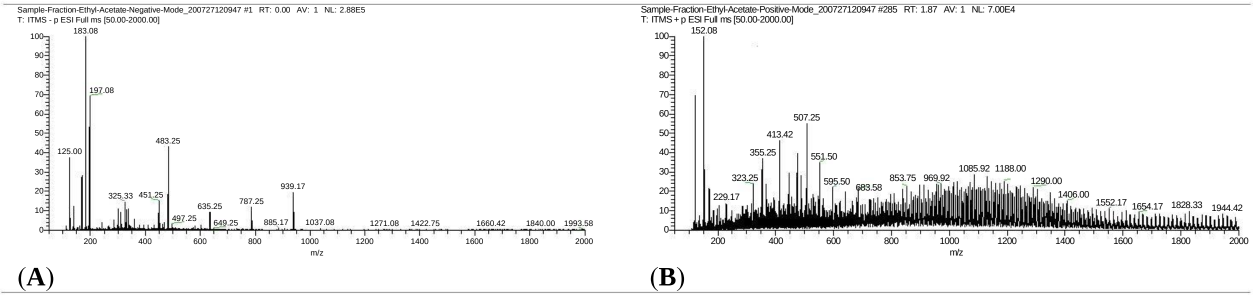

Figure S1. Full-scan ESI-LCMS chromatogram of the EtOAc fraction of *S.edelbergii*. (**A**) Negative ionization mode and (**B**) positive ionization mode.

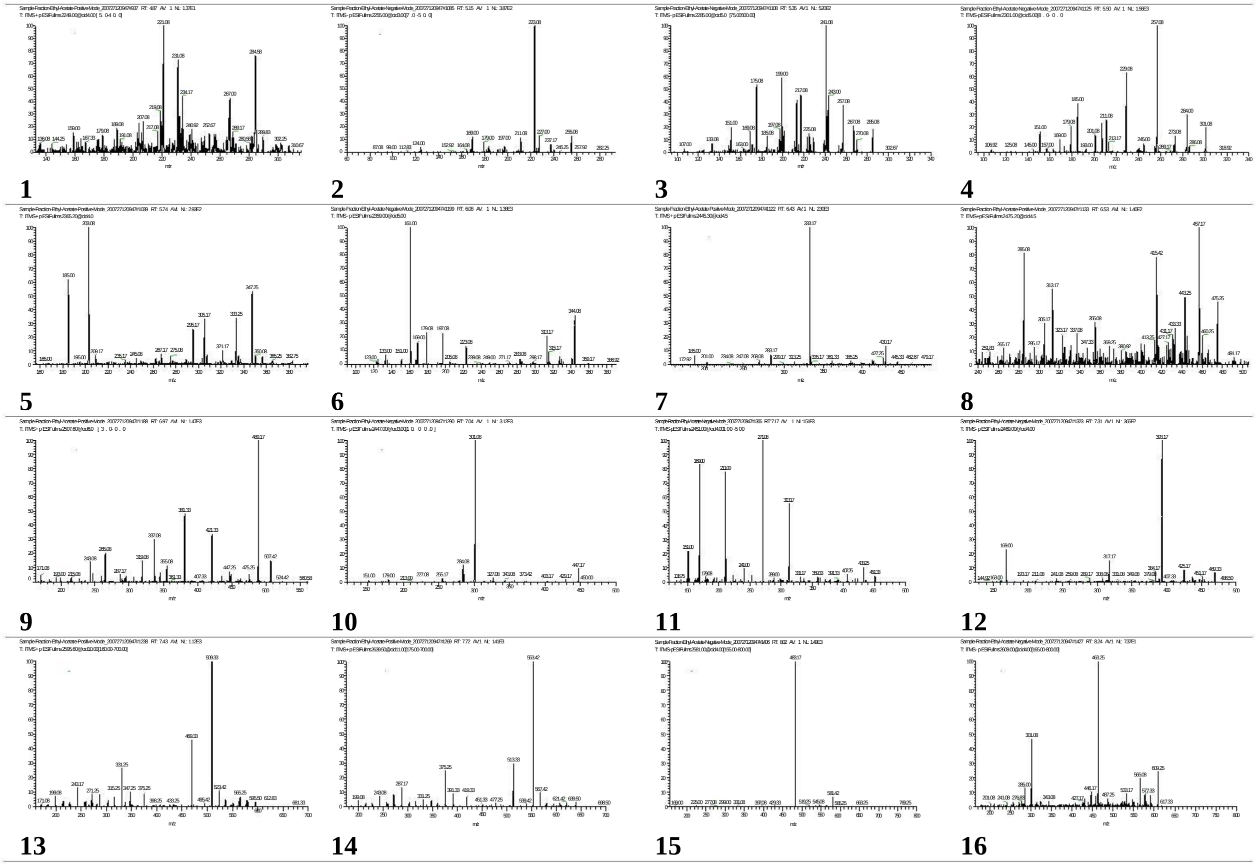

Figure S2. Chromatograms of ESI-LCMS identified compounds in the EtOAc fraction of *S.edelbergii*.
